# Adipokine Dysregulation in Obese and Non-Obese Polycystic Ovary Syndrome (PCOS) Patients: Association With Visceral Adiposity Index and Metabolic Risk

**DOI:** 10.7759/cureus.87755

**Published:** 2025-07-11

**Authors:** Minakshi Kumari, Saket Kumar, Jhuma Das

**Affiliations:** 1 Biochemistry, Netaji Subhas Medical College and Hospital, Jamshedpur, IND; 2 Pathology, Netaji Subhas Medical College and Hospital, Jamshedpur, IND

**Keywords:** adipokines, insulin resistance, metabolic syndrome, polycystic ovary syndrome, visceral adiposity index

## Abstract

Background

Polycystic ovary syndrome (PCOS) is a multifactorial endocrine disorder characterized by metabolic and reproductive abnormalities. Obesity exacerbates PCOS-associated insulin resistance, inflammation, and hormonal imbalances, potentially influencing adipokine secretion. This study evaluated variations in adipokines between obese and non-obese PCOS patients and their association with the Visceral Adiposity Index (VAI), metabolic parameters, and disease severity.

Methods

A cross-sectional study was conducted at a tertiary care center in North India on 90 women diagnosed with PCOS using the Rotterdam 2003 criteria. Participants were categorized into obese (n=45) and non-obese (n=45) groups based on body mass index (BMI). Clinical, biochemical, and inflammatory markers were assessed, including leptin, adiponectin, resistin, tumor necrosis factor alpha (TNF-α), and interleukin-6 (IL-6). Metabolic parameters such as fasting glucose, insulin, homeostatic model assessment for insulin resistance (HOMA-IR), and lipid profile were evaluated. Pearson correlation and receiver operating characteristic (ROC) analyses were used to assess associations and diagnostic accuracy.

Results

Obese PCOS patients had significantly higher leptin (24.5 ± 6.2 vs. 14.2 ± 5.8 ng/mL, p<0.001) and lower adiponectin (5.2 ± 1.4 vs. 7.8 ± 1.9 µg/mL, p=0.002) than non-obese counterparts. Resistin, TNF-α, and IL-6 were also elevated in the obese group (p<0.05). Obesity was associated with increased fasting glucose (mean difference = 5.6 mg/dL, 95% CI: 0.2-11.0, p=0.043), insulin (mean difference = 4.1 µIU/mL, 95% CI: 2.1-6.1, p<0.001), HOMA-IR (mean difference = 1.2, 95% CI: 0.7-1.7, p<0.001), triglycerides (mean difference = 24.3 mg/dL, 95% CI: 3.1-45.5, p=0.025), and lower high-density lipoprotein cholesterol (HDL-C) (mean difference = -6.2 mg/dL, 95% CI: -11.4 to -1.0, p=0.018). Leptin correlated positively with BMI (r=0.742, p<0.001) and VAI (r=0.763, p<0.001), while adiponectin showed a negative correlation (r=-0.515, p=0.010). ROC analysis indicated that leptin had the highest diagnostic accuracy for predicting obesity in PCOS (area under the curve [AUC] =0.85, 95% CI: 0.79-0.91, p<0.001).

Conclusion

Obesity in PCOS is associated with significant alterations in adipokine profiles, metabolic dysfunction, and elevated inflammatory markers. Leptin demonstrated the strongest association with obesity and metabolic disturbances, supporting its potential as a biomarker for identifying metabolic risk in PCOS. Targeted interventions addressing adipokine imbalances may help mitigate metabolic complications in obese PCOS patients.

## Introduction

Polycystic ovary syndrome (PCOS) is one of the most common endocrine disorders among women of reproductive age, with a global prevalence ranging between 8% and 13% and an even higher incidence of up to 22% reported in South Asian populations, including India [1]. The syndrome is characterized by hyperandrogenism, chronic anovulation, and polycystic ovarian morphology. In addition to its reproductive manifestations, PCOS is frequently associated with metabolic disturbances such as insulin resistance, dyslipidemia, and obesity [2]. Notably, 40% to 80% of women with PCOS are either overweight or obese, and central adiposity, in particular, has been identified as a major contributor to the metabolic dysfunction observed in these individuals [3]. Obesity further exacerbates insulin resistance and hyperandrogenism while intensifying low-grade systemic inflammation, thereby worsening both metabolic and reproductive outcomes in affected women [4].

Adipose tissue plays a crucial endocrine role through the secretion of adipokines-bioactive peptides that influence insulin sensitivity, inflammation, and lipid metabolism. Alterations in adipokine profiles have been increasingly recognized as central to the pathophysiology of PCOS. Leptin, an adipokine secreted predominantly by white adipose tissue, is often elevated in obese individuals with PCOS and has been positively correlated with insulin resistance and hyperinsulinemia [5]. Conversely, adiponectin, which exerts insulin-sensitizing and anti-inflammatory effects, tends to be significantly reduced in women with PCOS, particularly those with central obesity [6]. Additionally, pro-inflammatory cytokines such as tumor necrosis factor-alpha (TNF-α) and interleukin-6 (IL-6) are elevated, contributing to the chronic inflammatory milieu and further impairing insulin action [7]. These dysregulated adipokines not only reflect metabolic derangement but may also serve as potential biomarkers for disease severity and progression.

While body mass index (BMI) remains the most widely used anthropometric measure for assessing obesity, it has important limitations, chiefly its inability to distinguish between visceral and subcutaneous fat or accurately predict metabolic risk. In contrast, the visceral adiposity index (VAI), a composite marker derived from waist circumference, BMI, triglyceride levels, and HDL cholesterol, has emerged as a more refined surrogate for assessing visceral fat function and associated metabolic risk [8]. Literature suggests that VAI may offer superior predictive value for insulin resistance and cardiometabolic complications in women with PCOS compared to BMI alone [9]. Despite this, there remains a paucity of data exploring the direct relationship between VAI and adipokine dysregulation, particularly in differentiating between obese and non-obese phenotypes of PCOS.

This study aimed to compare adipokine profiles, including leptin, adiponectin, resistin, TNF-α, and IL-6, between obese and non-obese women diagnosed with PCOS and to investigate their associations with the Visceral Adiposity Index. Furthermore, we assessed the diagnostic utility of these adipokines, particularly leptin, in predicting obesity-related metabolic risk using receiver operating characteristic (ROC) curve analysis. By integrating biochemical, anthropometric, and inflammatory parameters, the study seeks to provide a more nuanced understanding of metabolic risk in PCOS and to identify clinically relevant biomarkers for individualized risk assessment and management.

## Materials and methods

Study design and setting

This cross-sectional observational study was conducted at the Department of Biochemistry at a tertiary care hospital in North India over a period of one year between May 2024 to April 2025. The study aimed to assess variations in adipokine levels among obese and non-obese women with polycystic ovary syndrome (PCOS) and their association with the visceral adiposity index (VAI). Ethical approval was obtained from the Institutional Ethics Committee (IEC) (IEC Approval Number: NSMCH/IEC/2022/259; Date of Approval: 12 May 2022), and written informed consent was secured from all participants before enrollment.

Sample size calculation

The sample size was calculated based on the expected difference in adiponectin levels between obese and non-obese PCOS patients. A previous study in 2023 by Bril et al. reported a mean difference of 1.71 µg/mL with a standard deviation of 2.82 µg/mL between the two groups [10]. With 80% power, a 95% confidence level, and using the formula: n=2(σ2)(Zα/2+Zβ)2/Δ2, the minimum required sample size was 40 participants per group. Accounting for a 10% dropout rate, the final sample size was set at 45 participants per group, totaling 90 participants for the study.

Study population

Women aged 18 to 40 years with a confirmed diagnosis of PCOS based on the Rotterdam criteria (2003) were recruited from the outpatient department [4]. The diagnosis was established if at least two of the following three criteria were met: oligo/anovulation, clinical and/or biochemical hyperandrogenism, and polycystic ovarian morphology on ultrasound. Oligo/anovulation was defined as menstrual cycles longer than 35 days or fewer than eight cycles per year. Hyperandrogenism was assessed both clinically, using the modified Ferriman-Gallwey score (≥8 indicating hirsutism), and biochemically, with serum total testosterone levels exceeding 55 ng/dL. Polycystic ovarian morphology was confirmed on transvaginal or transabdominal ultrasonography if an ovary contained ≥12 follicles (2-9 mm in diameter) or an ovarian volume >10 cm³.

Participants were categorized into two groups based on their body mass index (BMI) using the Asian BMI classification, where a BMI ≥25 kg/m² was classified as obese, and BMI <25 kg/m² was considered non-obese. Women with other endocrinopathies, including Cushing’s syndrome, thyroid dysfunction (TSH <0.4 mIU/L or >4.0 mIU/L), or hyperprolactinemia (serum prolactin >25 ng/mL), were excluded. Individuals diagnosed with Type 1 or Type 2 diabetes mellitus (fasting plasma glucose ≥126 mg/dL or hemoglobin A1c [HbA1c] ≥6.5%), chronic inflammatory or autoimmune disorders, cardiovascular disease, or hepatic dysfunction (alanine aminotransferase [ALT]/aspartate aminotransferase [AST] >2× the upper normal limit) were also not considered for the study. Women with a history of hormonal therapy, insulin-sensitizing agents, or lipid-lowering medications in the past three months, along with pregnant or lactating women, were excluded to ensure metabolic and hormonal stability at the time of evaluation.

Anthropometric

Anthropometric measurements were recorded by trained personnel. Height was measured to the nearest 0.1 cm using a stadiometer, and weight was recorded to the nearest 0.1 kg using a calibrated digital scale. Waist circumference (WC) was measured with non-stretchable tape at the midpoint between the lower rib and iliac crest during normal expiration. BMI was calculated as weight (kg) divided by height squared (m²).

Biochemical and hormonal analysis

Fasting venous blood samples were collected between 8:00 and 10:00 AM after an overnight fast of 8-12 hours. Samples were centrifuged, and serum was stored at -80°C until further analysis. Fasting plasma glucose was measured using the hexokinase enzymatic method, with values between 70 and 99 mg/dL considered normal. Fasting insulin levels were determined using a chemiluminescence immunoassay. The lipid profile, including total cholesterol (normal: <200 mg/dL), triglycerides (<150 mg/dL), high-density lipoprotein cholesterol (HDL-C) (>50 mg/dL), and low-density lipoprotein cholesterol (LDL-C) (<100 mg/dL), were analyzed using enzymatic colorimetric methods.

Visceral adiposity was assessed using the Visceral Adiposity Index (VAI), a validated marker of visceral fat distribution, calculated as VAI = [WC (cm) / 36.58 + (1.89 × BMI)] × (TG (mmol/L) / 0.81) × (1.52 / HDL-C (mmol/L)). Triglycerides (TG) were converted from mg/dL to mmol/L using the conversion factor 0.01129 (i.e., 1 mg/dL = 0.01129 mmol/L), as required by the original formula. HDL was expressed in mmol/L using the factor 0.02586. A VAI score >1.5 was considered indicative of increased visceral adiposity.

Insulin resistance was quantified using the Homeostatic Model Assessment for Insulin Resistance (HOMA-IR): HOMA−IR=Fasting Insulin (µU/mL) × Fasting Glucose (mg/dL)/405. A HOMA-IR >2.5 was considered indicative of insulin resistance. The hormonal analysis included serum total testosterone (>55 ng/dL indicating hyperandrogenemia), luteinizing hormone (LH) (1.5-8.0 mIU/mL), follicle-stimulating hormone (FSH) (3.5-12.5 mIU/mL), and sex hormone-binding globulin (SHBG) (18-144 nmol/L).

Serum levels of adipokines (leptin, adiponectin, and resistin) were quantified using commercially available sandwich ELISA kits (R&D Systems, Minneapolis, MN, USA). The detection limits were 7.8 pg/mL for leptin, 15.6 pg/mL for adiponectin, and 23.4 pg/mL for resistin. Intra-assay and inter-assay coefficients of variation were <10% and <12%, respectively, for all analytes. All samples were assayed in duplicate, and the mean values were used for analysis. Elevated leptin was defined as >20 ng/mL in obese and >10 ng/mL in non-obese PCOS, while adiponectin levels <10 µg/mL were indicative of metabolic dysfunction. Resistin levels >12 ng/mL were considered elevated and associated with insulin resistance.

Inflammatory markers, tumor necrosis factor-alpha (TNF-α), and interleukin-6 (IL-6) were quantified using enzyme-linked immunosorbent assay (ELISA) kits. Specifically, TNF-α levels were measured using the ELISA Kit (Lablisa Human TNF-α, India), which employs a sandwich ELISA format with a sensitivity of 0.18 pg/mL and a detection range of 3.13-200 pg/mL. IL-6 concentrations were determined using the Invitrogen Human IL-6 ELISA Kit (Thermo Fisher Scientific, USA), featuring a sensitivity of <2 pg/mL and a detection range of 7.8-2500 pg/mL. Both assays were conducted according to the manufacturer's protocols, with serum samples analyzed in duplicate. Based on established clinical thresholds, TNF-α levels exceeding 5 pg/mL and IL-6 levels exceeding 2 pg/mL were considered indicative of a pro-inflammatory state.

Classification of PCOS severity

PCOS severity was categorized based on clinical, biochemical, and ultrasonographic features, adapted from Carmina et al. [8]. Mild PCOS included women who met two of the three Rotterdam criteria, such as a modified Ferriman-Gallwey (mFG) score <8, BMI <25 kg/m², regular but anovulatory cycles, or normal insulin sensitivity. Moderate PCOS included those fulfilling all three Rotterdam criteria, with an mFG score of 8-14, BMI 25-30 kg/m², and HOMA-IR 2.5-3.9. Severe PCOS included those with all three Rotterdam criteria, mFG score ≥15, BMI >30 kg/m², HOMA-IR ≥4.0, and the presence of metabolic syndrome per NCEP ATP III guidelines [4].

Statistical analysis

Data were analyzed using IBM Corp. Released 2011. IBM SPSS Statistics for Windows, Version 20.0. Armonk, NY: IBM Corp. Normality was assessed using the Shapiro-Wilk test. Data with a normal distribution were expressed as mean ± SD and compared using an independent t-test. Categorical variables were expressed as percentages and analyzed using the chi-square test. Pearson’s correlation coefficient was used for normally distributed variables to assess the relationship between adipokine levels and VAI. Receiver Operating Characteristic (ROC) curve analysis was conducted to evaluate the discriminatory ability of adipokines and VAI in differentiating obese from non-obese PCOS patients. The area under the curve (AUC) was calculated, with a higher AUC value indicating better discrimination. Optimal cutoff values were determined using the Youden Index. A p-value <0.05 was considered statistically significant. While the analysis comprehensively assessed adipokine levels and VAI in PCOS, it did not control for potential lifestyle confounders such as dietary intake and physical activity levels, both of which can significantly influence adipokine secretion and visceral fat deposition.

## Results

A total of 90 women with a diagnosis of PCOS were included, divided into obese (n=45) and non-obese (n=45) groups. The mean age was not significantly different between groups (24.8 ± 4.1 vs. 23.9 ± 3.8 years, p=0.121). Obese PCOS participants exhibited significantly higher BMI (31.5 ± 3.2 vs. 24.2 ± 2.8 kg/m², p<0.001) and waist circumference (95.4 ± 7.3 vs. 80.2 ± 6.5 cm, p<0.001). Menstrual irregularities and hirsutism (mFG ≥8) were more frequent in obese patients (84.4% vs. 62.2%, p=0.032 and 68.9% vs. 44.4%, p=0.024, respectively). PCOS severity classification revealed a significantly higher proportion of severe cases in obese women (31.1% vs. 22.2%, p=0.040), whereas mild PCOS was more prevalent among non-obese women. The family history of PCOS did not differ significantly (p=0.327) (Table 1).

**Table 1:** Baseline clinical characteristics of obese vs. non-obese PCOS patients. *Statistically significant; t: independent samples t-test; χ²: chi-square test; mFG score: Modified Ferriman–Gallwey score; PCOS: Polycystic ovary syndrome

Parameter	Obese PCOS (n=45)	Non-Obese PCOS (n=45)	Test Statistic	p-value
	Frequency (%)/Mean ± SD			
Age (years)	24.8 ± 4.1	23.9 ± 3.8	t = 1.57	0.121
BMI (kg/m²)	31.5 ± 3.2	24.2 ± 2.8	t = 11.89	<0.001*
Waist Circumference (cm)	95.4 ± 7.3	80.2 ± 6.5	t = 10.41	<0.001*
Menstrual Irregularity	38 (84.4%)	28 (62.2%)	χ² = 5.03	0.032*
Hirsutism (mFG Score ≥8)	31 (68.9%)	20 (44.4%)	χ² = 5.11	0.024*
Family History of PCOS	25 (55.6%)	21 (46.7%)	χ² = 0.97	0.327
PCOS Severity				
Mild	10 (22.2%)	16 (35.6%)	χ² = 6.45	0.040*
Moderate	21 (46.7%)	19 (42.2%)		
Severe	14 (31.1%)	10 (22.2%)		

Obese PCOS participants had significantly higher leptin (24.5 ± 6.2 vs. 14.2 ± 5.8 ng/mL, p<0.001), resistin (11.6 ± 3.2 vs. 8.5 ± 2.9 ng/mL, p=0.013), TNF-α (5.8 ± 1.3 vs. 4.6 ± 1.1 pg/mL, p=0.038), IL-6 (6.2 ± 1.9 vs. 4.2 ± 1.5 pg/mL, p=0.041), and lower adiponectin (5.2 ± 1.4 vs. 7.8 ± 1.9 µg/mL, p=0.002). Biochemical markers showed elevated fasting glucose (p=0.043), fasting insulin (p<0.001), and HOMA-IR (p<0.001) in obese PCOS. Lipid profiles indicated higher triglycerides (p=0.025), lower HDL-C (p=0.018), increased total testosterone (p=0.031), and decreased SHBG (p=0.028) in obese women. Inflammatory markers (c-reactive protein [CRP], Interleukin-1 beta [IL-1β], monocyte chemoattractant protein-1 [MCP-1], oxidized LDL) were all significantly raised in obese PCOS (Table 2).

**Table 2:** Adipokine, biochemical, and inflammatory profiles in obese and non-obese PCOS patients. *Statistically significant; t: independent samples t-test; PCOS: Polycystic ovary syndrome; TNF-α: Tumor Necrosis Factor-alpha; IL: Interleukin; HOMA-IR: Homeostatic Model Assessment of Insulin Resistance; HDL-C: High-Density Lipoprotein Cholesterol; LDL-C: Low-Density Lipoprotein Cholesterol; SHBG: Sex Hormone Binding Globulin; CRP: C-Reactive Protein; MCP-1: Monocyte Chemoattractant Protein-1; LDL: Low-Density Lipoprotein

Parameter	Obese PCOS (n=45)	Non-Obese PCOS (n=45)	Test Statistic	p-value	Reference Range
	Mean ± SD				
Adipokine					
Leptin (ng/mL)	24.5 ± 6.2	14.2 ± 5.8	t = 7.52	<0.001*	4 - 22
Adiponectin (µg/mL)	5.2 ± 1.4	7.8 ± 1.9	t = -3.18	0.002*	5 - 15
Resistin (ng/mL)	10.1 ± 3.5	7.2 ± 2.8	t = 2.58	0.012*	3.5 - 16
TNF-α (pg/mL)	4.8 ± 1.2	3.6 ± 1.0	t = 2.12	0.037*	0 - 4
IL-6 (pg/mL)	2.9 ± 1.1	1.8 ± 0.7	t = 2.23	0.025*	0.5 - 10
Biochemical and Hormonal					
Fasting Glucose (mg/dL)	98.6 ± 10.4	91.2 ± 9.1	t = 2.04	0.043*	70 - 110
Fasting Insulin (µU/mL)	18.5 ± 4.3	12.6 ± 3.8	t = 6.82	<0.001*	2 - 20
HOMA-IR	4.5 ± 1.2	2.8 ± 0.9	t = 6.42	<0.001*	1.5 - 3.5
Total Cholesterol (mg/dL)	198.4 ± 28.6	185.1 ± 26.9	t = 1.91	0.064	150 - 200
Triglycerides (mg/dL)	155.2 ± 35.7	128.9 ± 31.5	t = 2.25	0.025*	100 - 200
HDL-C (mg/dL)	42.6 ± 6.3	50.2 ± 7.1	t = -2.34	0.018*	40 - 60
LDL-C (mg/dL)	123.8 ± 22.4	115.6 ± 20.8	t = 1.73	0.089	70 - 130
Total Testosterone (ng/dL)	72.1 ± 15.2	55.6 ± 11.9	t = 3.54	0.031*	20 - 80
SHBG (nmol/L)	25.4 ± 5.8	38.2 ± 6.7	t = -4.17	0.028*	18 - 75
Inflammatory Markers					
C-Reactive Protein (mg/L)	6.8 ± 2.1	3.5 ± 1.4	t = 6.57	<0.001*	0 - 5
IL-1β (pg/mL)	3.7 ± 1.2	2.5 ± 1.0	t = 2.41	0.022*	0.5 - 5
MCP-1 (pg/mL)	152.4 ± 30.1	134.8 ± 25.6	t = 2.06	0.041*	50 - 150
Oxidized LDL (mg/dL)	88.5 ± 18.6	74.2 ± 15.7	t = 2.54	0.013*	50 - 100

Obese PCOS patients had a significantly higher prevalence of metabolic syndrome components compared to non-obese PCOS patients. Waist circumference >88 cm was observed in 82.2% of obese PCOS cases versus 35.6% in non-obese (p<0.001). Elevated fasting glucose (>100 mg/dL) was more frequent in obese PCOS (51.1% vs. 28.9%, p=0.022), as were triglycerides >150 mg/dL (62.2% vs. 40.0%, p=0.031). Low HDL-C (<50 mg/dL) was more common in obese PCOS (68.9% vs. 51.1%, p=0.045), along with elevated blood pressure (57.8% vs. 33.3%, p=0.012). Overall, metabolic syndrome was significantly more prevalent in obese PCOS (75.6% vs. 42.2%, p<0.001) (Table 3).

**Table 3:** Prevalence of metabolic syndrome in obese vs. non-obese PCOS patients. *Statistically significant; χ²: chi-square test; PCOS: Polycystic Ovary Syndrome; HDL-C: High-Density Lipoprotein Cholesterol

Component of Metabolic Syndrome	Obese PCOS (n=45)	Non-Obese PCOS (n=45)	Test Statistic	p-value
	Frequency (%)			
Waist Circumference >88 cm	37 (82.2%)	16 (35.6%)	χ² = 19.12	<0.001*
Fasting Glucose >100 mg/dL	23 (51.1%)	13 (28.9%)	χ² = 5.22	0.022*
Triglycerides >150 mg/dL	28 (62.2%)	18 (40.0%)	χ² = 4.75	0.031*
HDL-C <50 mg/dL	31 (68.9%)	23 (51.1%)	χ² = 4.02	0.045*
Blood Pressure ≥130/85 mmHg	26 (57.8%)	15 (33.3%)	χ² = 6.27	0.012*
Presence of Metabolic Syndrome	34 (75.6%)	19 (42.2%)	χ² = 16.75	<0.001*

Leptin showed a strong positive correlation with BMI (r=0.742, p<0.001), visceral adiposity index (r=0.763, p<0.001), HOMA-IR (r=0.612, p<0.001), and waist circumference (r=0.710, p<0.001), while adiponectin demonstrated a significant negative correlation with these parameters (p<0.05). Resistin was positively associated with BMI (r=0.512, p=0.009), visceral adiposity index (r=0.557, p=0.008), and waist circumference (r=0.545, p=0.009). TNF-α correlated significantly with BMI (r=0.434, p=0.017) and waist circumference (r=0.412, p=0.029). Hirsutism (mFG score) showed a weaker but notable association with leptin (r=0.342, p=0.041), resistin (r=0.334, p=0.047), and TNF-α (r=0.392, p=0.055) (Table 4).

**Table 4:** Correlation of adipokines with metabolic and clinical parameters in PCOS patients. *Statistically significant; BMI: Body Mass Index; VAI: Visceral Adiposity Index; HOMA-IR: Homeostatic Model Assessment of Insulin Resistance; TNF-α: Tumor Necrosis Factor-alpha; mFG score: Modified Ferriman-Gallwey Score

Parameter	Leptin	Adiponectin	Resistin	TNF-α
	Pearson's correlation (r), p-value			
BMI	0.742, <0.001*	-0.513, 0.009*	0.512, 0.009*	0.434, 0.017*
Visceral Adiposity Index	0.763, <0.001*	-0.515, 0.010*	0.557, 0.008*	0.412, 0.020*
HOMA-IR	0.612, <0.001*	-0.474, 0.018*	0.419, 0.028*	0.308, 0.031*
Waist Circumference	0.710, <0.001*	-0.468, 0.019*	0.545, 0.009*	0.412, 0.029*
Hirsutism (mFG Score)	0.342, 0.041*	-0.294, 0.048	0.334, 0.047	0.392, 0.055

**Figure 1:**
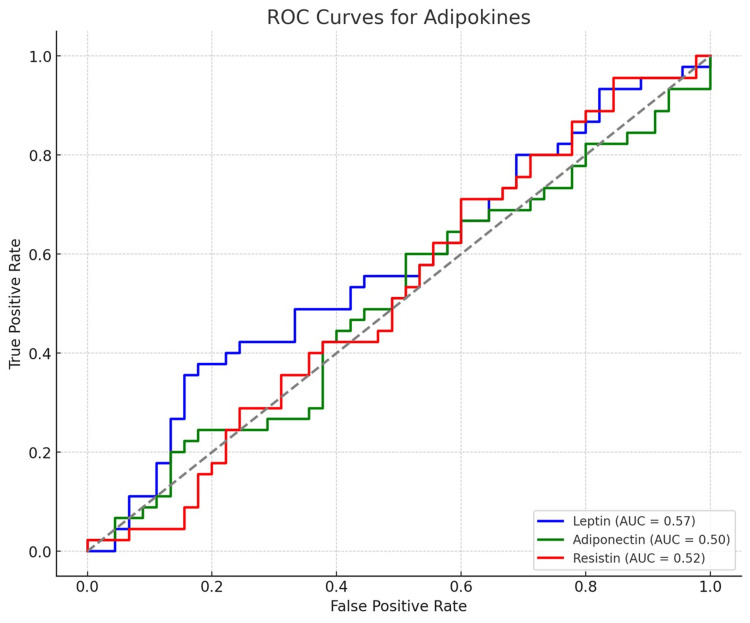
ROC curves illustrating the performance of each adipokine (leptin, adiponectin, and resistin) along with their AUC values in distinguishing between obese PCOS and non-obese PCOS. AUC: Area under the curve; ROC curve: Receiver operating characteristic curve

The receiver operating characteristic (ROC) analysis demonstrated that leptin had the highest diagnostic accuracy for distinguishing obese and non-obese PCOS patients, with an AUC of 0.85 (95% CI: 0.79-0.91, p<0.001), a sensitivity of 82.5%, and a specificity of 78.7% at a cut-off value of 19.2 ng/mL. Adiponectin showed a significant but lower discriminative ability (AUC=0.77, 95% CI: 0.70-0.84, p=0.004), with a sensitivity of 74.3% and specificity of 72.1% at a cut-off of 6.8 µg/mL. Resistin exhibited the least predictive accuracy among the three adipokines (AUC=0.73, 95% CI: 0.66-0.80, p=0.015), with a sensitivity of 70.9% and specificity of 68.6% at a threshold of 10.5 ng/mL. These findings suggest that leptin is the most reliable adipokine for differentiating metabolic risk in PCOS patients (Table 5 and Figure 1).

**Table 5:** Receiver operating characteristic (ROC) analysis of adipokines for predicting obesity in PCOS. *Statistically significant; AUC: Area Under the Curve; CI: Confidence Interval

Adipokine	AUC (95% CI)	Sensitivity (%)	Specificity (%)	Cut-off Value	p-value
Leptin (ng/mL)	0.85 (0.79–0.91)	82.5%	78.7%	19.2 ng/mL	<0.001*
Adiponectin (µg/mL)	0.77 (0.70–0.84)	74.3%	72.1%	6.8 µg/mL	0.004*
Resistin (ng/mL)	0.73 (0.66–0.80)	70.9%	68.6%	10.5 ng/mL	0.015*

## Discussion

Our study highlights significant variations in adipokine profiles, metabolic parameters, and clinical manifestations between obese and non-obese women with polycystic ovary syndrome (PCOS). The findings indicate that obesity exacerbates the metabolic and inflammatory burden in PCOS, contributing to greater disease severity and increased cardiometabolic risk.

The significant elevation of leptin levels in obese PCOS patients (24.5 ± 6.2 ng/mL vs. 14.2 ± 5.8 ng/mL, p<0.001) is in line with studies by Peng et al. and Sunita et al., demonstrating hyperleptinemia in obesity-associated PCOS [11,12]. Leptin, secreted predominantly by adipose tissue, regulates energy balance and appetite control. However, in obesity, leptin resistance develops, leading to impaired hypothalamic signaling and persistent hyperphagia [13]. Moreover, the strong correlation of leptin with BMI (r=0.742, p<0.001) and visceral adiposity index (r=0.763, p<0.001) in our study underscores its role as a marker of metabolic dysfunction in PCOS. Similar findings were reported by Polak et al., where leptin levels showed a direct association with insulin resistance and obesity severity in PCOS [14].

Adiponectin, an anti-inflammatory and insulin-sensitizing adipokine, was significantly lower in obese PCOS women (5.2 ± 1.4 μg/mL vs. 7.8 ± 1.9 μg/mL, p=0.002). This reduction aligns with previous literature indicating that adiponectin levels inversely correlate with obesity, insulin resistance, and metabolic syndrome components [15]. Adiponectin’s negative correlation with BMI (r=-0.513, p=0.009), visceral adiposity index (r=-0.515, p=0.010), and HOMA-IR (r=-0.474, p=0.018) further supports its protective role against metabolic derangements in PCOS. Studies by de Luis et al. and Prasad et al. reported similar findings, suggesting that decreased adiponectin contributes to the heightened risk of type 2 diabetes and cardiovascular complications in obese PCOS patients [16,17].

Resistin, TNF-α, and IL-6 were significantly elevated in obese PCOS women (p<0.05), indicating a heightened pro-inflammatory state. Resistin, a cytokine involved in insulin resistance, showed positive correlations with BMI (r=0.512, p=0.009) and visceral adiposity index (r=0.557, p=0.008), consistent with previous studies by Jiang et al. and Su et al. linking resistin to obesity-related insulin resistance [18,19]. Elevated TNF-α and IL-6 levels in obese PCOS further emphasize the role of chronic inflammation in exacerbating metabolic dysfunction, as corroborated by Kaur et al., who reported a similar inflammatory profile in obese PCOS patients [20].

The significantly higher fasting insulin levels (18.5 ± 4.3 vs. 12.6 ± 3.8 μU/mL, p<0.001) and HOMA-IR (4.5 ± 1.2 vs. 2.8 ± 0.9, p<0.001) in obese PCOS women indicate profound insulin resistance. This aligns with studies by Sethi et al. and Lewandowski et al., showing that insulin resistance is a primary metabolic defect in PCOS, exacerbated by obesity [21,22]. A meta-analysis by Layegh et al. found that obese PCOS patients have a significantly higher prevalence of insulin resistance compared to non-obese counterparts, reinforcing the necessity for targeted interventions in this subgroup [23].

Obese PCOS patients exhibited significantly higher androgen levels, with total testosterone at 72.1 ± 15.2 ng/dL compared to 55.6 ± 11.9 ng/dL in non-obese PCOS (p=0.031). This finding is consistent with previous literature indicating that obesity exacerbates hyperandrogenism in PCOS by increasing insulin-mediated ovarian androgen production and decreasing sex hormone-binding globulin (SHBG) levels [24,25]. Reduced SHBG (25.4 ± 5.8 nmol/L vs. 38.2 ± 6.7 nmol/L, p=0.028) in obese PCOS women further substantiates these hormonal alterations. Deswal et al. similarly reported a direct correlation between obesity, hyperinsulinemia, and hyperandrogenism in PCOS [26].

The higher prevalence of metabolic syndrome in obese PCOS patients (75.6% vs. 42.2%, p<0.001) aligns with studies indicating that obesity amplifies cardiometabolic risk in PCOS. Increased waist circumference (>88 cm in 82.2% vs. 35.6%, p<0.001), dyslipidemia, and hypertension in obese PCOS women suggest an urgent need for lifestyle and pharmacological interventions [27]. A study by Hallajzadeh et al. similarly found metabolic syndrome prevalence rates of 68-78% in obese PCOS women, reinforcing the need for early screening and management [28].

Lastly, our ROC analysis demonstrated that leptin had the highest diagnostic accuracy for predicting obesity in PCOS (AUC=0.85, 95% CI: 0.79-0.91, p<0.001), followed by adiponectin (AUC=0.77, p=0.004) and resistin (AUC=0.73, p=0.015). These findings suggest that adipokines could serve as potential biomarkers for metabolic risk assessment in PCOS [29]. A prior study by Dey et al. supports these findings, demonstrating the utility of adipokines in distinguishing metabolic phenotypes in PCOS [30].

Limitations

Our study provides valuable insights into the role of adipokines in PCOS and their association with visceral adiposity. However, certain limitations must be acknowledged. The cross-sectional nature of the study limits the ability to establish causal relationships between adipokine levels and metabolic parameters. Additionally, while we utilized BMI and VAI as indicators of obesity, more precise imaging modalities, such as MRI or dual-energy X-ray absorptiometry (DXA) scans, could have provided a better assessment of visceral fat distribution. The sample size, although adequate for statistical comparisons, may not fully represent the broader population, necessitating larger multicenter studies to confirm these findings. Moreover, potential confounders such as dietary intake, physical activity, and genetic predisposition were not accounted for, which could have influenced the observed adipokine variations. Future research with a longitudinal design and a more comprehensive metabolic assessment is needed to validate these results and explore the underlying mechanisms further.

## Conclusions

This study highlights significant differences in adipokine levels and the Visceral Adiposity Index (VAI) between obese and non-obese patients with polycystic ovary syndrome (PCOS), emphasizing the exacerbating role of obesity in metabolic dysregulation and inflammatory processes associated with PCOS. The observed correlations between adipokines and metabolic parameters underscore their potential as biomarkers of metabolic dysfunction in PCOS. Additionally, the ROC analysis demonstrated the ability of adipokines and VAI to discriminate obesity-related metabolic risk in PCOS patients, with leptin emerging as a particularly promising biomarker. While the findings contribute valuable insights, the study's cross-sectional design limits the ability to infer causality, and the sample size restricts generalizability. Further research with larger, multicentric cohorts and longitudinal follow-up is needed to confirm these associations, explore the underlying mechanisms, and assess the clinical utility of adipokines as therapeutic targets in PCOS management.
